# Association of GRM7 Variants with Different Phenotype Patterns of Age-Related Hearing Impairment in an Elderly Male Han Chinese Population

**DOI:** 10.1371/journal.pone.0077153

**Published:** 2013-10-11

**Authors:** Huajie Luo, Tao Yang, Xiaojie Jin, Xiuhong Pang, Jiping Li, Yongchuan Chai, Lei Li, Yi Zhang, Luping Zhang, Zhihua Zhang, Wenjing Wu, Qin Zhang, Xianting Hu, Jingwen Sun, Xuemei Jiang, Zhuping Fan, Zhiwu Huang, Hao Wu

**Affiliations:** 1 Department of Otolaryngology Head and Neck Surgery, Xinhua Hospital, Shanghai JiaoTong University School of Medicine, Shanghai, China; 2 Ear Institute, Shanghai JiaoTong University School of Medicine, Shanghai, China; 3 Health check-up center, Xinhua Hospital, Shanghai JiaoTong University School of Medicine, Shanghai, China; 4 Department of Otolaryngology Head and Neck Surgery, RenJi Hospital, Shanghai JiaoTong University School of Medicine, Shanghai, China; 5 Health check-up center, RenJi Hospital, Shanghai JiaoTong University School of Medicine, Shanghai, China; University of Texas School of Public Health, United States of America

## Abstract

Several single nucleotide polymorphisms (SNPs) of the Glutamate metabotrophic receptor 7 gene (*GRM7*) have recently been identified by the genome-wide association study (GWAS) as potentially playing a role in susceptibility to age-related hearing impairment (ARHI), however this has not been validated in the Han Chinese population. The aim of this study was to determine if these SNPs are also associated with ARHI in an elderly male Han Chinese population. In this case-control candidate genes association study, a total of 982 men with ARHI and 324 normal-hearing controls subjects were studied. Using K-means cluster analysis, four audiogram shape subtypes of ARHI were identified in the case group: ‘‘flat shape (FL)’’, ‘‘sloping shape (SL)’’, ‘‘2-4 kHz abrupt loss (AL) shape’’ and ‘‘8 kHz dip (8D) shape’’. Results suggested that the SNP rs11928865 (A>T) of *GRM7* was significantly associated with ARHI after adjusting for non-genetic factors (p= 0.000472, OR= 1.599, 95%CI= 1.229~2.081). Furthermore, frequency of TT genotype (rs11928865) were significant higher in the SL subgroup and AL subgroup with compared to controls group (p= 9.41E-05, OR= 1.945, 95%CI= 1.393~2.715; p= 0.000109, OR= 1.915, 95%CI= 1.378~2.661 adjusted, respectively) after Bonferroni correction. However, there wasn’t significant difference in the frequency of the TT genotype between cases in the FL subgroup or the 8D subgroup with when compared with controls. Results of the current study suggest that, in an elderly male Han Chinese population, *GRM7* SNP rs11928865 (TT) occurs more frequently in ARHI patients with SL and AL phenotype patterns.

## Introduction

Age-related hearing impairment (ARHI), also known as presbycusis, is a multifactorial symmetric sensorineural loss that affects adults older than 50 years of age [1]. According to the World Health Organization’s (WHO) world health statistics report 2012, the average life expectancy in China has increased from 68 years in 1990 to 74 years of age in 2009, [2]. The prevalence of ARHI is increasing at an alarming rate due to the aging population and is becoming a major sensory problem among the elderly [3-5]. Previous studies have clearly shown a heritability of approximately 0.5 for ARHI [6-10], however the genetic susceptibility to ARHI has not been clarified until recently.

Methodologically, two of the most powerful strategies for identifying susceptibility genes are linkage analyses and association studies [11,12]. Three linkage studies of ARHI have been previously published [13-15], although with those negative results. Association studies have attempted to identify genetic variations that occur more frequently in unrelated affected compared to unrelated, unaffected individuals [11,12]. The potassium voltage-gated channel member 4 gene (*KCNQ4*,OMIM ID:603537) [16]; N-acetyltransferases (NAT2*6A,OMIM ID:612182) [17-19]; the grainyhead-like 2 gene (*GRHL2* ,OMIM ID: 608576) [20]; the apolipoprotein E gene (*APOE ε4* OMIM ID: 107741) [21]; the endothelin-1 gene (*EDN1*, OMIM ID: 131240) [22,23]; the uncoupling protein gene (*UCP2*, OMIM ID: 601693) [24] and the mitochondrial DNA (mtDNA) 4977 common deletion [25,26] have all been reported to be correlated with ARHI. Compared to candidate genes association studies, the genome-wide association study (GWAS) might provide the most convincing evidence for genetic susceptibility to complex diseases. Three GWASs of ARHI have been published recently [14,27,28]. Research done by Friedman et al. [27] provided convincing evidence that variations in glutamate metabotrophic receptor 7 (*GRM7*, OMIM ID: 604101) was associated with ARHI in older European adults. The top-ranked SNP, rs11928865, was associated with ARHI and was localized in intron 2 of the *GRM7* gene on chromosome 3. The fine mapping of the *GRM7* locus in the European replication group demonstrated that rs11928865 remained the most significantly associated individual SNP, while haplotype blocks 6,7 (consisting of SNPs rs6804466, rs3828472, rs9819783, rs11920109, rs11928865 and rs9877154) were the most significantly associated haplotype blocks [27]. Newman et. al [29] also explored the relationship of the *GRM7* haplotypes and SNP genotypes with various measures of auditory perception in a European-American population. In another GWAS study, a Finnish Saami population was scrutinized for individuals with ARHI [28]. In this study, Van Laer et al. found a SNP locus, rs161927, downstream of *GRM7* (p= 0.000149) that correlated with ARHI pure-tones audiometric data. Unfortunately, similar research has not yet been reported in the Asian Han population.

Clinically, pure-tone audiometry is the gold standard for measuring hearing impairment [30-32]. Research into the genetics of ARHI done by Friedman et al [27] has focused solely on hearing as measured by pure-tone thresholds based on the Z-score method [33], although there are several inevitable shortcomings. The Z-score expresses the difference of the median value for a particular age and gender in standard deviation units based on ISO 7029 standards [34], however the current ISO standard does not include subjects over 70 years of age. Another potential issue with this method is that, in previous studies, ARHI audiogram patterns were difficult to distinguish since subjects with the best (controls) and worst (cases) Z-score hearing results were selected. 

 To reduce the multivariate phenotype of ARHI, while capturing most of the phenotypic pattern variation and still retaining biologically important features of the audiogram shapes, Cheng-Yung Lee [35] designed a statistical classification system of audiogram shapes in order to improve and integrate shape recognition across clinical settings. K-means cluster analysis was employed to categorize audiometric shapes. Using this analysis method, similar patterns, shared by homogeneous subjects, can be grouped and the dissimilar patterns from heterogeneous subjects can be separated. The classification of audiogram shapes is expected to provide better guidelines and greater accuracy when diagnosing ARHI.

The aim of this study was to verify GRM7 variants previously reported to assess the impact on the risk of ARHI in an elderly male Han population over 70-years of age. The association of GRM7 polymorphisms with different audiogram phenotype patterns of ARHI categorized via K-means cluster analysis will also be discussed.

## Materials and Methods

The Institutional Review Ethics boards of Shanghai Jiatong University School of Medicine, Xinhua Hospital and RenJi Hospital approved the study protocol. The protocol was in compliance with the Declaration of Helsinki and informed written consent was obtained from all participants.

Subjects for this case controlled study were recruited from the same geographic region (Shanghai) and were of the same ethnic origin (HAN population) but no subject was related to any other subject in the study. Adult male volunteers, aged 70 to 100 years, were recruited between July 2011 and December 2012 from the health check-up centers of Xinhua and Renji Hospitals, which are affiliated with the Shanghai Jiatong University School of Medicine. Health check-up centers offer routine, annual health examinations for geriatric individuals. These examinations include a short questionnaire of systemic disease history and health behavior(smoking, alcohol consumption) and a basic physical examination including a chest X-ray, electrocardiography, and blood biochemistry tests.

Information regarding hearing loss history, including exposure to noise and ototoxic drug exposure was collected from study participants. An otoscopic examination was then conducted to exclude any ear pathology that could potentially affecting hearing. Audiologic results were measured with air and bone conduction thresholds of pure tones, using an audiometer (TDH39 earphone, Madsen Itera Inc, Taastrup, DENMARK.). Air conduction thresholds were assessed at 0.25, 0.5, 1, 2, 4, 6 and 8 kHz and bone conduction was assessed at 0.5, 1, 2, and 4 kHz. Pure-tone thresholds were measured in one ear at a time, in a quiet room, and a blood sample was taken from each subject at the time of testing.

### 1: The criteria for selection

Individuals with symmetric sensorineural hearing loss (pure-tone threshold average (PTA) of 0.5 kHz, 1 kHz, 2 kHz, and 4 kHz more than 25 dB HL) were screened and included the ARHI case group for genotyping and further analysis. Table S1 summarizes the strict diagnostic criteria for the ARHI case group. Individuals with a PTA less than 25 dB HL in both ears were included in the healthy control group.

### 2: Audiogram data transformation and K-means cluster analysis

K-means cluster analysis was employed, as described by Cheng-Yung Lee [35], to categorize the audiometric shapes in the better ear of individuals in the ARHI case subjects group. To ensure baseline homogeneity, all ARHI shapes were calibrated to zero dB at 0.25 kHz; the other frequencies were then adjusted to the difference from the 0.25 kHz threshold. Shapes with different thresholds, but with the same configuration, were categorized into one subtype shape by K-means cluster analysis using SPSS version 19.0.

The exact number of audiogram shapes was observed in a stepwise fashion. That is, cluster analysis was put forward from 2 clusters, and the clustered results were obtained using ANOVA. By doing this, there was enough heterogeneity between the final number of categorized audiogram shapes,but homogeneity within a specific clustered shape. The optimal end-point for cluster analysis was satisfied statistically using maximal F statistics and minimal mean square errors. This provided an explanation of the similarities and dissimilarities among the clustered shapes. An important consideration for shape recognition was to match configurations of octave frequencies after minimizing within-shape variation and maximizing between-shape variation. The number of shapes was then tested for statistical and clinical meaningfulness.

### 3: SNP selection and Genotyping

**Figure 1:**
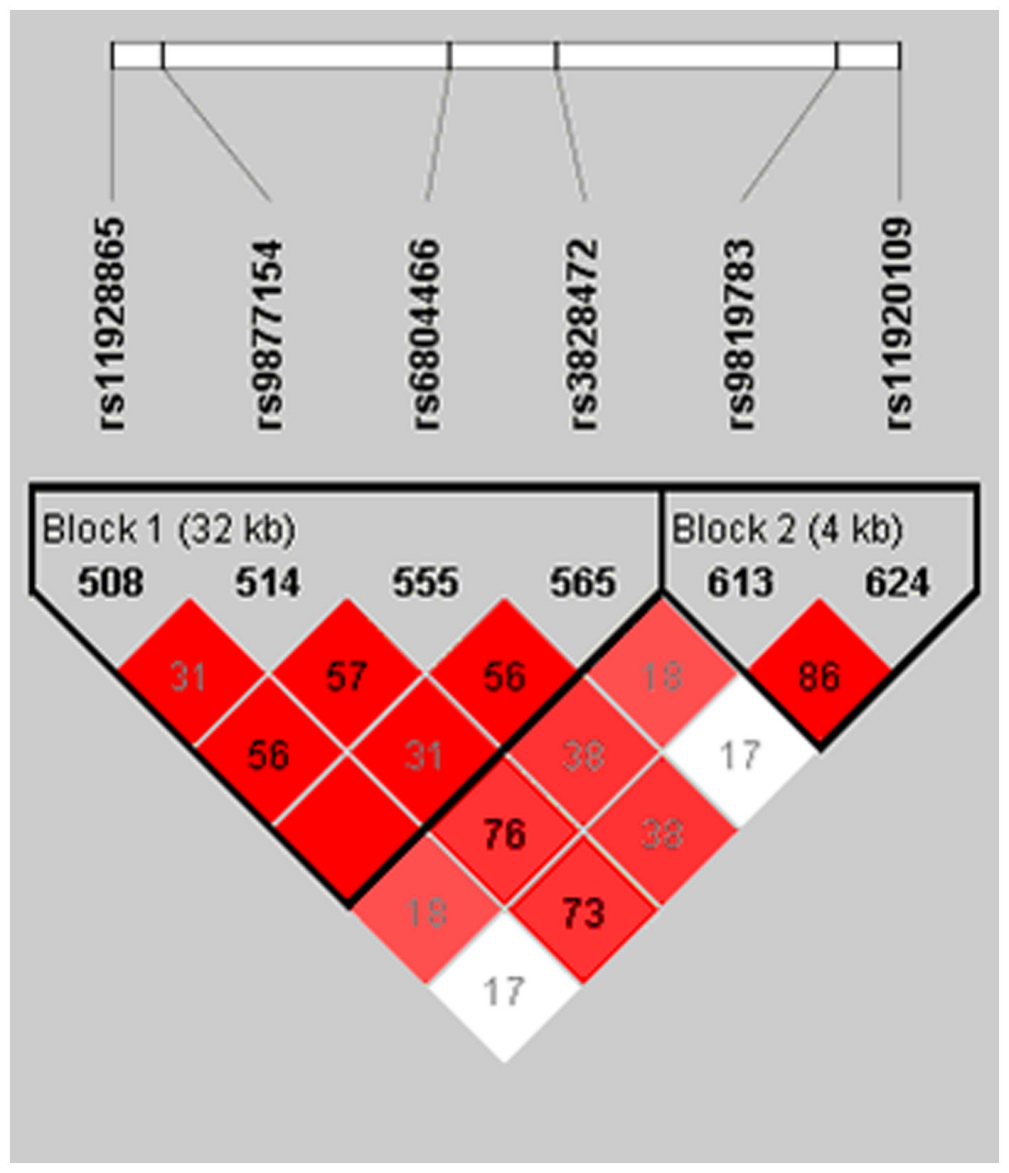
Linkage disequilibrium (LD) across SNPs selected of the *GRM7* gene. The results of LD mapping were generated using Haploview software. The value for r^2^ between each selected SNP is presented in each box. Red boxes denote strong LD (D’= 1.0) and white boxes denote very weak LD (r^2^= 0.173,).

Based on HapMap data Rel 27 PhaseII+III,Feb09,on NCBI B36 assembly, dbSNP b126 (http://hapmap.ncbi.nlm.nih.gov/cgi-perl/gbrowse/hapmap27_B36/#search), the *GRM7* single nucleotide polymorphisms (SNPs: rs11928865, rs9877154, rs6804466, rs3828472, rs9819783, rs11920109) associated with ARHI, that were identified by Friedman [27] in European sample group, were also found to be present in CHB population dbSNP database. The results of linkage disequilibrium (LD) mapping were generated using Haploview software 4.2 (shown in Figure 1). SNPs rs11928865, rs9877154, rs6804466, and rs3828472 were present in haploblock 1 and SNPs rs9819783, and rs11920109 belonged to haploblock 2. Therefore, two taqSNP loci were selected, rs11928865 and rs11920109 (one from haploblocks 1 and 2, respectively r^2^= 0.173,D’= 0.815, LOD= 1.85), to examine and verify the correlation between *GRM7* SNPs and genetic susceptibility to ARHI in the male Chinese Han population.

Genotyping analyses were performed on the selected *GRM7* SNPs (rs11928865: A>T, rs11920109: C>T) using PCR amplification and direct sequencing in the Molecular Biology of Hearing and Deafness Research Laboratory, Ear Institute, Shanghai Jiaotong University. Genomic DNA was extracted from whole blood samples using a standard extraction method. The SNP loci were then amplified using by nest PCR. Primer sequences for *GRM7* analysis are shown in Table S2. The 25μL PCR reactions were done in a 2720 Thermal Cycler (Applied Biosystems, Foster City, CA, USA). The PCR cycling conditions included an initial denaturation step at 94°C for 5 minutes followed by 33 cycles of denaturation at 94°C for 30 seconds, annealing at 55°C for 45 seconds, extension at 72°C for 45 seconds and a final extension step at 72°C for 7 minutes. Sequencing of the product after PCR was performed with an ABI PRISM 3730xl Genetic Analyzer (Applied Biosystems).

### 4: Statistical Analysis

The data are presented as mean±standard deviation unless indicated otherwise. Each SNP was tested using a chi-squared test for Hardy Weinberg Equilibrium. Genotype frequency distribution and allele frequency between ARHI patients and healthy controls was tested using the Chi square test or Fisher’s exact test. Association of SNPs with ARHI was further confirmed by logistic regression analysis after adjusting for possible confounding factors: Central obesity (CO),and cardiovascular disease (CAD), hypertension (HTN), diabetes mellitus (DM), dyslipidemia (DL),chronic kidney disease (CKD), chronic obstructive pulmonary disease (COPD), anemia, osteoporosis (OP), smoking, drinking. Odds ratios (OR) and 95% confidence intervals (CI) were used to analyze the occurrence of the high-risk genotypes in the Chinese Han population sample. For regression modeling in the additive model, homozygotes for the major allele (1/1), and heterozygotes (1/0) and homozygotes for the minor allele (0/0) were coded to an ordered categorical variable for the genotype (0, 1 and 2). The dominant model was defined as 0/0, 1/0 versus 1/1 and the recessive model as 0/0 versus 1/0, 1/1. The level of significance was set at p≤0.05. we used a Bonferroni correction to correct for the effects of multiple comparisons. All calculations were performed with the statistical software package 19.0.0 (IBM SPSS Statistics, Inc., Chicago, IL).

### 5: Power of Study

Power estimation is an important part of a genetic association study and is used to identify candidate genes that contribute to disease susceptibility. Power calculations for the current study were done using QUANTO software version 1.2 4 (http://hydra.usc.edu/gxe/) with the following options: an unmatched case-control study design, a population risk of 40% [37], a significance level of 0.05, a T-allele frequency of 0.78 for rs11928865 and an inheritance recessive genetic mode. Based on our data, the sample size of 982 subjects in the case group vs. 324 in the control group that was included in our study had a power of 92.8% to detect an association (OR _adjusted_ = 1.599). Sample sizes of 354 in the AL subgroup vs. 324 in the control group and 337 in the SL subgroup vs. 324 in the control group had powers of 98.1% and 98.4%, (OR _adjusted_= 1.915 and 1.945), respectively, to detect an association.

## Results

A total of 1306 male volunteers met the selection criteria: 324 subjects were assigned to the healthy control group, and 982 individuals were deemed eligible for ARHI case group in this study.

The mean age for the control group was 80.31±5.37 years (range 71–94 years) and the pure-tone threshold average in the better ear was 21.23±2.84 dB HL at 0.5, 1, 2, and 4kHz. The mean age of the ARHI case group was 80.84±5.45 years (range 71–100 years) and the pure-tone threshold average in the better ear was 44.02±13.52 dB HL at 0.5, 1, 2, and 4kHz. There was no significant difference in age between the ARHI case and control groups.

### 1: Audiogram phenotype pattern Classification

**Figure 2:**
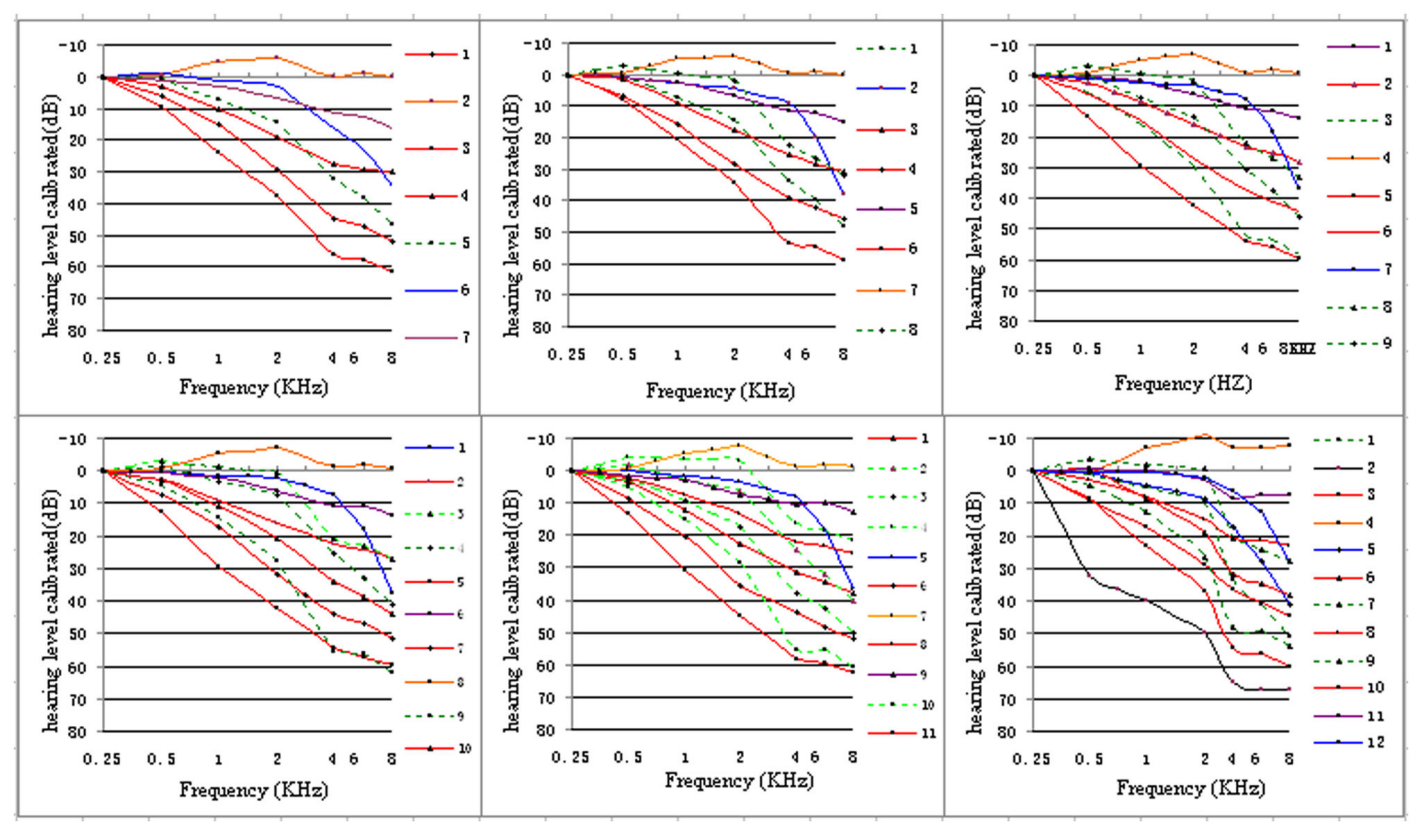
Illustrates shape 7-,8-, 9-, 10-, 11- and 12- solutions of ARHI group audiogram patterns classified by k-means cluter analysis. For easy comparison, all audiometric shapes are calibrated at the left most frequency (0.25 kHz). Basic audiogram shapes from seven-shape solution, appear. Three lines: the flat shape (blue solid line, yellow solid line) and 8 kHz dip (8D) shape (purple solid line), and is almost identical to 11-shape. The sloping shape group (red solid line) and brutal loss group (green dotted line) are very uniform and easy to distinguish in the 11-shape solution. In the twelve-shape solution, the new shape (black line 2) appears and was observed in a small group of 2 subjects.

Table S3 illustrates the stepwise analysis of K-means cluster analysis from audiogram shapes 4 through 13 in the ARHI case subjects group. Figure 2 illustrates shape 7-,8-, 9-, 10-, 11- and 12- solutions. The pattern solution in Figure 2 revealed a preliminary structure of shapes beginning at the eighth shape. Using the standard classification system and nomenclature AMCLASS^TM^ [36], the three graphics presented: the flat (FL) shape (yellow solid line, purple solid line) and the 8 kHz dip (8D) shape (blue solid line) are very stable.

The solution was examined up to 1 shape 2 since no other meaningful clinical shapes were observed beyond that point. One shape (black solid line) was observed in only 2 subjects. In other words, a unique shape was derived that was difficult to separate into smaller groups. 

The ANOVA was done using shape solutions 7 through 12 (Table S4). Mean square errors were used to form and differentiate the clusters of the shapes and the F statistic was helpful in determining the ideal number of shapes. When examining the most widely distributed variation in the presbycusis audiogram, the F statistics of the 2, 4, 6,and 8 kHz frequencies was weighted more than the F statistics of the 0.5 and 1 kHz frequencies. According to the standard classification system and nomenclature, AMCLASS^TM^, typical abrupt loss graphics and sloping graphics were clearly subgrouped, in shapes 8 - 10 and especially in shape 11. The abrupt loss graphics group (green dotted line) and the sloping graphics group (red solid line) were easy to distinguish and were subgrouped. Moreover, the F statistics at 2, 4, 6, and 8 kHz in the 12 shapes were less than of the F statistics at the same points in shape 11 and the mean square error at 2, and 6 kHz of 12 shapes was larger than that of the shape 11. However, the mean square error of all 2, 4, 6, and 8 kHz and the F statistic at 8 kHz in 10 shapes could be enough against that in 11 shapes. Thus, shape 11, rather than shape 12 or 10, was chosen according to end point criteria. Therefore, based on this database, the ideal statistical and clinical number of classified shapes was 11.

Table 1 illustrates the nomenclature and subgroup of 11 audiogram shapes used in the current study Based on the AMCLASS^TM^ standard description [36],each of the subgroup shapes identified in this study can be conclusively described for clinical applications. The 11 audiogram shapes are described as follows:

**A flat (FL**)** shape** is an audiogram where all octave thresholds fluctuate within ±10 dB difference to the mean (yellow solid line 7, purple solid line 9).

**A 2-4kHz abrupt loss (AL**)** shape** is an audiogram where a difference of more than 20 dB exists between 2kHz and 4kHz (green dotted line 8, 1, 6, 11).

**An 8 kHz dip (8D**)** shape** is an audiogram where the 8 kHz threshold is poorer by more than 20 dB than others (blue solid line 5). 

**A sloping (SL**)** shape** is an audiogram where higher frequency thresholds are worse than lower frequency thresholds by 10–40 dB (red solid line 4, 2, 3, 10).

**Table 1:** Nomenclature and subgroups of audiogram 11 shapes.

Nom of configuration	Audiogram line number	n(%)	Total n(%)
Flat shape(FL)	7	66(6.72%)	200(20.37%)
	9	134(13.65%)	
8 kHz dip (8D) shape (8D)	5	91(9.27%)	91(9.27%)
2-4kHz abrupt loss shape(AL)	4	68(6.92%)	354(36.05)
	2	136(13.85%)	
	3	94(9.57%)	
	10	56(5.70%)	
Sloping shape (SL)	8	142(14.46%)	337(34.32%)
	1	110(11.20%)	
	6	68(6.92%)	
	11	17(1.73%)	
Total in case group			982(100%)

### 2: Genotyping

Allele and genotype frequencies of these SNPs loci were compatible with Hardy – Weinberg equilibrium in both case and control subjects. Association study between the selected taqSNPs and the ARHI in case and control groups are shown in Table 2: The Gene polymorphisms at GRM7 (rs11920109) in subjects with ARHI was not significantly different than in controls (*P*_freq_= 0.438, OR_Unadjusted_= 1.074, 95%CI= 0.896~1.287; *P*_freq_= 0.303,). However, the genotype distribution of *GRM7* SNP rs11928865 in the ARHI case group was significantly different than in controls (*P*_freq_= 0.000524, OR_Unadjusted_= 1.482, 95%CI= 1.185~1.852). Four subtypes of audiogram shape 11 were determined using K-means cluster analysis as previously described (Table 1). Gene polymorphisms at *GRM7* rs11928865 were analyzed in each subgroup. Table 3 shows the distribution of the *GRM7* rs11928865 genotype polymorphisms in each ARHI subgroup and the control group. The T-allele frequency of SNP rs11928865 (*GRM7*) was 0.78 in the control group. The T-allele frequencies of rs11928865 were 0.863 and 0.862 in the sloping (SL) shape group and 2-4kHz abrupt loss (AL) shape group, respectively and were significantly different from those of the control group (P<0.05). The T-allele frequencies of rs11928865 in the flat (FL) shape and 8 kHz dip (8D) shape audiogram subgroups were not significantly different when compared to that of the control group.

**Table 2:** Association study between the selected taqSNPs and ARHI in Case and Control Groups.

**S**NPs	**Genotype distribution**					**Allele frequencies**					**OR**_Unadjusted_ (**95%CI**)
		Control(n)	%	ARHI(n)	%		Control(n)	%	ARHI(n)	%	
rs11928865	AA	11	3.40%	19	1.93%	A	140	21.60%	308	15.68%	1.482(1.185-1.852)
	AT	118	36.42%	270	27.49%	T	508	78.40%	1656	84.32%	*P*_freq_= 0.000524
	TT	195	60.19%	693	70.57%						
rs11920109	CC	114	35.19%	332	33.81%	C	392	60.49%	1142	58.15%	1.074(0.896-1.287)
	CT	164	50.62%	478	48.68%	T	256	39.51%	822	41.85%	*P*_freq_= 0.438
	TT	46	14.20%	172	17.52%						

OR, odds ratio; CI, confidence interval

**Table 3:** Association of *GRM7* SNP rs11928865 in patients with different audiogram patterns of ARHI (based on the K-means cluster analysis) and in control subjects.

Group		Genotype		P for Genotype			Allele		P for Allele	OR for T Allele (95%CI)
	AA	AT	TT	Dominant model	additive model	recessive model	A	T		
							minor allele	major allele		
Control subjects (n=324 )										
Count	11	118	195				140	508		
Frequency (%)	3.40%	36.42%	60.19%				21.60%	78.40%		
All case patients(n=982)				0.12817	0.00178	0.00051			0.00100	1.482(1.185-1.852)
Count	19	270	693				308	1656		
Frequency (%)	1.93%	27.49%	70.57%				15.68%	84.32%		
FL subgroup case patients(n=200)				0.05169	0.89450	0.70260			0.66400	0.936(0.694-1.262)
Count	8	75	117				91	309		
Frequency (%)	4.00%	37.50%	58.50%				22.75%	77.25%		
SL subgroup case patients(n=337)				0.18986	0.00058	0.00013			0.00020	1.722(1.291-2.296)
Count	6	81	250				93	581		
Frequency (%)	1.78%	24.04%	74.18%				13.80%	86.20%		
AL subgroup case patients(n=354)				0.04518	0.00041	0.00017			0.00013	1.736(1.306-2.307)
Count	4	89	261				97	611		
Frequency (%)	1.13%	25.14%	73.73%				13.70%	86.30%		
8D subgroup case patients(n=91)				0.24810	0.11195	0.05010			0.04400	1.582(1.009-2.480)
Count	1	25	65				27	155		
Frequency (%)	1.10%	27.47%	71.43%				14.84%	85.16%		

OR, odds ratio; CI, confidence interval

Logistic regression analyses were constructed to control for nongenetic, confounding variables. The recessive model of genotype tests was the best fitting model for the logistic regression analyses (Table 3). The dependent variable in the logistic regression models was the group classification (case or control), with the case group coded as 1 and the control group coded as 0. The independent variables included the *GRM7* genotype, central obesity (CO), cardiovascular disease (CAD), hypertension (HTN), diabetes mellitus (DM), dyslipidemia (DL), chronic kidney disease (CKD), chronic obstructive pulmonary disease (COPD), anemia, osteoporosis (OP), smoking, and alcohol consumption. The adjusted ORs for *GRM7* rs11928865 genotypes TT vs AA+AT for different audiogram patterns in ARHI are shown in Table 4. After adjusting for other risk factors, the OR of the genotype TT was significantly different in the ARHI compared with the control group (*P* adjusted= 0.000472, OR adjusted= 1.599, 95%CI= 1.229~2.081). The adjusted OR of genotype TT was significantly different in both the SL and AL audiogram pattern subgroups compared with the control group (*P*_adjusted_= 9.41E-05, OR_adjusted_= 1.945, 95%CI= 1.393~2.715, *P*_adjusted_= 0.000109, OR_adjusted_= 1.915, 95%CI= 1.378~2.661 respectively). The *P* value remained significant even after applying the Bonferroni correction (*P*_adjusted_ < 0.0125 (0.05/4)). The adjusted OR of the TT genotype was not significantly different from the control group in either the FL or the 8D audiogram pattern subgroups (*P*_adjusted_ =0.708209, OR_adjusted_= 0.933, 95%CI= 0.647~1.344, and *P*_adjusted_= 0.071177, OR adjusted= 1.607, 95%CI= 0.960~2.690, respectively).

**Table 4:** Logistic Regression Model: analysis of the effects of *GRM7* SNP rs11928865 genotypes and nongenetic factors in case and control groups.

	Control subjects (n=324 )														
	VS														
	All case patients(n=982)			AL subgroup case patients(n=354)			8D subgroup case patients(n=91)			SL subgroup case patients(n=337)			FL subgroup case patients(n=200)		
Variable	P	OR	95%CI	P	OR	95%CI		OR	95%CI	P	OR	95%CI	P	OR	95%CI
AA+AT	-	-		-	-	-	-	-	-	-	-	-	-	-	-
TT	0.000472	1.599	1.229 ~ 2.081	0.000109	1.915	1.378 ~ 2.661	0.071177	1.607	0.960 ~ 2.690	9.41E-05	1.945	1.393 ~ 2.715	0.708209	0.933	0.647 ~ 1.344
CO	0.670	0.946	0.735 ~ 1.219	0.284	0.845	0.621 ~ 1.150	0.677	0.904	0.562 ~ 1.454	0.310	0.851	0.623 ~ 1.162	0.108	1.345	0.937 ~ 1.930
CAD	0.976	1.003	0.805 ~ 1.251	0.862	1.020	0.819 ~ 1.270	0.817	0.934	0.521 ~ 1.673	0.510	0.881	0.605 ~ 1.284	0.629	1.108	0.730 ~ 1.683
HTN	0.830	1.029	0.795 ~ 1.331	0.871	0.974	0.710 ~ 1.336	0.218	1.359	0.834 ~ 2.213	0.814	0.962	0.700 ~ 1.323	0.521	1.127	0.782 ~ 1.625
DM	0.348	1.157	0.853 ~ 1.569	0.681	1.081	0.746 ~ 1.567	0.578	0.845	0.467 ~ 1.530	0.139	1.319	0.914 ~ 1.902	0.534	1.144	0.749 ~ 1.748
DL	0.636	1.073	0.802 ~ 1.436	0.427	1.154	0.811 ~ 1.640	0.645	1.137	0.659 ~ 1.959	0.585	1.104	0.773 ~ 1.577	0.763	0.937	0.616 ~ 1.426
CKD	0.956	1.010	0.700 ~ 1.458	0.813	0.946	0.600 ~ 1.492	0.978	0.990	0.502 ~ 1.954	0.518	1.158	0.742 ~ 1.809	1.000	1.000	0.597 ~ 1.675
COPD	0.566	0.927	0.715 ~ 1.201	0.515	0.907	0.677 ~ 1.216	0.795	1.048	0.737 ~ 1.488	0.664	0.937	0.700 ~ 1.255	0.844	0.969	0.710 ~ 1.324
Anemia	0.934	0.992	0.815 ~ 1.206	0.626	0.953	0.786 ~ 1.156	0.874	0.979	0.755 ~ 1.270	0.851	0.982	0.811 ~ 1.188	0.542	1.062	0.875 ~ 1.290
0P	0.706	0.952	0.736 ~ 1.231	0.536	0.906	0.664 ~ 1.237	0.415	1.225	0.752 ~ 1.995	0.757	0.952	0.695 ~ 1.304	0.661	1.085	0.753 ~ 1.564
Smoking	0.647	0.940	0.721 ~ 1.225	0.091	0.760	0.553 ~ 1.045	0.594	1.152	0.684 ~ 1.942	0.593	0.915	0.660 ~ 1.268	0.570	1.116	0.764 ~ 1.632
Drinking	0.771	0.960	0.731 ~ 1.262	0.485	0.889	0.638 ~ 1.237	0.063	1.732	0.971 ~ 3.088	0.300	0.837	0.597 ~ 1.173	0.782	1.057	0.713 ~ 1.568

Adjusted Odds Ratios for genotypes TT vs. AA+AT of *GRM7* SNP rs11928865 for ARHI in different audiogram patterns (by Logistic Regression Analysis under the recessive mode) in case and control groups.

ORs adjusted for other clinical traits: central obesity (CO), cardiovascular disease (CAD), hypertension (HTN), diabetes mellitus (DM), dyslipidemia (DL), chronic kidney disease (CKD), chronic obstructive pulmonary disease (COPD), anemia, osteoporosis (OP).

Subsequent analysis of the other taqSNPs selected (rs11920109), did not show any significant associations when comparing any of the four subtypes in ARHI case group with the healthy control group.

## Discussion

It is extremely important to conduct studies in subjects that have actual ARHI, as it is well known that environmental noise and ototoxic agents can influence hearing in the elderly. A difference due to gender has also been universally acknowledged to be a major factor that influences ARHL. Hearing loss due to age tends to happen faster, occur earlier and the loss tends to be more severe in men than in women [8,38-40]. Thus, environmental factors must be strictly excluded in order to avoid confusion and interference by confounding variables when researching possible genetic factors in ARHI. Studies need to be carefully designed and the influence of gender needs to be taken into account. Therefore, only male volunteers were included in the current study and the study was designed with strict inclusion and exclusion criteria.

There are a number of pathophysiological processes underlying age-related changes in the auditory system. Perception of sound involves many complex pathways and age-related changes in any one component of these pathway could contribute to ARHI. Therefore, the site of gene expression in the auditory pathways and whether the site of a lesion corresponds to a particular hearing phenotype need to be taken into account when selecting candidate genes for genetic susceptibility studies. 

 Schuknecht et al’s ARHI classification system was groundbreaking [41,42]. Studies of the cochlea in many animal species and histopathological studies of human temporal bones have shown that inner hair cell and outer hair cell, stria vascularis, spiral ganglion cells, as well as many other cochlear cell types and structures show age-related degeneration [43-45]. Schuknecht et al [41,42] proposed a correlation between audiogram pattern shapes and the lesion sites of ARHI. According to this classification, a flat loss (FL) audiogram pattern is related to the degeneration in the stria vascularis and a sloping (SL) or abrupt loss (AL) audiogram pattern is related to the degeneration of hair cells and spiral ganglion cells.

The protein product of *GRM7*, mGluR7,has been observed in inner and outer hair cells and in the spiral ganglion nerve cell bodies of the inner ear [27]. *GRM7* is thought to be central to maintaining glutamate synaptic transmission and homeostasis in the mammalian cochlea at the synapses between hair cells and in the dendrites of afferent auditory nerve fibers [27]. The presence of glutamate in excessive quantities has been suggested as a possible mechanism that mediates neurotoxicity in auditory neurons [46]. 

According to the opinion of Schuknecht [41,42], high-frequency hearing loss in ARHI, represented by the SL or AL audiogram shape, may be related to missing outer hair cells, degeneration of the 8th nerve, or stiffness properties of the basilar membrane. Results of the current study indicated that the T-allele frequencies of rs11928865 in the SL and AL subgroups were 0.863 and 0.862, respectively and compared to the genotype AA+AT, the adjusted ORs of genotypes TT was significantly different from controls in the SL and AL subgroups (*P* adjusted= 9.41E-05, OR adjusted= 1.945, 95%CI= 1.393~2.715, *P* adjusted= 0.000109, OR adjusted= 1.915, 95%CI= 1.378~2.661 respectively), even after applying a Bonferroni correction.

Similarly, according to Schuknecht, an FL audiogram indicates that the ARHI is mainly due to the degeneration of the strial vascularis based on Schuknecht opinion [41,42]. However, there is no evidence of GRM7 expression in the stria vascularis [27]. Our data showed that the GRM7 rs11928865 polymorphisms in the FL subgroup were not significantly different from those found in the controls group (*P*_adjusted_= 0.708209, OR_adjusted_= 0.933, 95%CI= 0.647~1.344). These data imply that there may be other genetic mechanisms involved in ARHI patients with flat audiogram shapes. In addition we found that the *GRM7* genetic distribution of the GRM7 rs11928865 polymorphism in the 8D subgroup was not significantly different when comparing ARHI patients to healthy controls (*P*_adjusted_= 0.071177, OR_adjusted_= 1.607, 95%CI= 0.960~2.690). However, in this case, the adjusted *P* value was nearly significant (*P*_adjusted_= 0.071177) so the relatively small sample size of 8D subgroup might be the main reason for the negative result.

Prior to categorizing the ARHI audiogram phenotype patterns into subgroups, we found that the distribution of the *GRM7* SNP rs11920109 was not significantly different in ARHI group compared to the healthy control group. However, the *GRM7* SNP rs11928865 (A>T) was significantly different in the ARHI compared with the control group. These findings are inconsistent with those reported by Friedman et al. One reason for this may be population differences since the Friedman study was conducted in a European-American population. Another reason that the findings of the current study differ from those of previous studies may be that different statistical methods were used to analyze the phenotype audiogram patterns in ARHI.

The Z-score method that was used in previous studies is based on International Standard (ISO) 7029 standards. This method converts frequency-specific thresholds to a gender- and age-independent value, that is referred to as the Z-score. The ISO 7029 describes the median (P50) threshold of hearing by air conduction as a function of age for men and women. The threshold data are mainly based on studies done in the 1950s, 1960s and 1970s [47]. There is a possibility that these data may be inaccurate due to outdated selection criteria and calibration procedures. The current ISO standard does not include subjects older than 70 years of age. Due to this restriction, case-control association studies of genetic susceptibility to ARHI rarely involve the elderly population (those over the age of 70). However, we believe that those over the age of 70 are likely to be more representative for research into ARHI genetic susceptibility for ARHI. There is great variation in the age of onset, progression and severity of ARHI, even though every individual shows a steady decline in hearing ability with age. Even if hearing is completely normal in an individual under the age of 70, it will be not necessarily still normal when the same individual is over the age of 70. In other words, it is possible that the control group also contained some patients with ARHI which may have lead to falsely-negative results. It is also difficult to distinguish truly different ARHI hearing phenotype patterns in ARHI using the Z-score method. Furthermore, there may be several different mechanisms responsible for the different hearing phenotype patterns in ARHI. Different audiogram patterns might confound the relationship between gene polymorphisms and ARHI: The GRHL2 gene has been associated with susceptibility to ARHI in a study by Van Laer et al. [20]. In that study, a very steeply sloping audiogram shape was predominantly observed. However, Lin YH et al. [48] reported another negative result of GRHL2 susceptibility with ARHI, the samples in this study having a higher proportion of flat-shaped audiograms might be also a reason of the different results in these two researchs.

The K-means cluster analysis in mathematical modeling and engineering applications has been widely applied. The algorithm based on the K value of a clustering algorithm, is an effective way to solve the clustering problem, provides a statistical classification system to categorize audiometric shape patterns of ARHI in those over 70 years of age and was particularly useful in our study. For conducting large sample audiological statistics, this method could help avoid potential mistakes that are made when audiologists artificially group audiograms by phenotype using professional experience rather than a less subjective method. Our results suggest that the accurate audiogram phenotypic grouping is necessary for in the candidate gene association studies of ARHI.

## Conclusions

Results of our study indicate that the K-means cluster analysis method can be used to subgroup ARHI hearing phenotype patterns. Based on the subgrouping phenotype, the *GRM7* SNP rs11928865 was only associated with ARHI in subjects with “sloping “and “abrupt loss” audiogram phenotype patterns in which anatomical function pathology was observed in the hair cells and in the spiral ganglion nerve cells. Future research into ARHI genetic susceptibility should take into account different hearing phenotype patterns. 

## Supporting Information

Table S1 **Exclusive diagnostic criteria of ARHL.**
(DOC)

Table S2 **Nest PCR primer sequences for GRM7 analysis.**
(DOC)

Table S3 **The number of ARHI case subjects group in each stepwise observation of K-means cluster analysis.**
(DOC)

Table S4 **ANOVA table of mean square error and F statistic in the ARHI case subjects group.**
(DOC)
