# Supplementary material for: Association of GRM7 Variants with Different Phenotype Patterns of Age-Related Hearing Impairment in an Elderly Male Han Chinese Population
Source: PLoS One. 2013 Oct 11;8(10):e77153. doi: 10.1371/journal.pone.0077153 (PMC3795658; doi:10.1371/journal.pone.0077153)
Supplement: Table S1 — Exclusive diagnostic criteria of ARHL. (DOC) [file pone.0077153.s001.doc]

**Table S1 : Exclusive diagnostic criteria of ARHL** [16][27][33][48]

| - **AUDIOLOGICAL AND CLINICAL OTOLOGICAL PATHOLOGIES** |
| --- |
| 1. **1.Clinical criteria**  - One or both tympanic membranes scored as acute otitis media, inactive chronic otitis media   or active chronic otitis   - Syndromic features of congenital otological abnormalities   **2.Sensorineural hearing impairment**   - Menières disease - VIII nerve tumor - Ramsay hunt syndrome - Post-meningitis - All sudden losses - Subjects with cochlear implant - Noise or ototoxic drug-induced HL - Other genetic hearing loss - Severe head injury   **3.Audiological criteria**   - Conductive hearing loss, air-bone gap averaged over 0.5,1,2 kHz of >15 dB in one or   both ears   - Unilateral or significantly asymmetric (greater than 25 dB difference in interaural pure-   tone average, PTA) hearing loss;   - Asymmetrical hearing loss, difference between left and right ear air conduction   thresholds of ≥ 20dB for at least 2 frequencies   - Noise dips were calculated as the difference between air thresholds at 4,000 and 8,000 Hz   and were excluded if they exceeded 20 dB.  **4.History criteria**   - Hearing loss developing before 30 years of age   Exposure of the head and neck to radiation |
| - **GENERAL PATHOLOGIES** - Designed by an international European consortium   (see details at http://www.interscience.wiley.com/jpages/1059-7794/suppmat) |
