# Supplementary material for: Association of GRM7 Variants with Different Phenotype Patterns of Age-Related Hearing Impairment in an Elderly Male Han Chinese Population
Source: PLoS One. 2013 Oct 11;8(10):e77153. doi: 10.1371/journal.pone.0077153 (PMC3795658; doi:10.1371/journal.pone.0077153)
Supplement: Table S2 — Nest PCR primer sequences for GRM7 analysis. (DOC) [file pone.0077153.s002.doc]

**Table S2: Nest PCR primer sequences for GRM7** analysis

| GRM7 SNP | Primers |  |  |
| --- | --- | --- | --- |
| rs11928865 | First pair of PCR primer | forward | 5' TCAACATATTGCCCAGGCTGG 3' |
|  |  | reverse | 5' AGGGACTGAGCATCTTCAG 3' |
|  | Second pair of PCR primer | forward | 5' ACAGGCATGACTCTTGATG 3' |
|  |  | reverse | 5' ATGTTAAGTAGCCTGACAGC 3' |
| rs11920109 | First pair of PCR primer | forward | 5' ATTCTGTATGACCTGGCTTCG 3' |
|  |  | reverse | 5' GAAAGTGGAAGTGTGTGGTGC 3' |
|  | Second pair of PCR primer | forward | 5' ATCTCCTTCCAGGCTCATCT 3' |
|  |  | reverse | 5' AACACAGACAAGCAGAGGGA 3' |
