# Supplementary material for: Association of GRM7 Variants with Different Phenotype Patterns of Age-Related Hearing Impairment in an Elderly Male Han Chinese Population
Source: PLoS One. 2013 Oct 11;8(10):e77153. doi: 10.1371/journal.pone.0077153 (PMC3795658; doi:10.1371/journal.pone.0077153)
Supplement: Table S3 — The number of ARHI case subjects group in each stepwise observation of K-means cluster analysis. (DOC) [file pone.0077153.s003.doc]

**Table S3. The number of ARHI case subjects group in each stepwise observation of K-means cluster analysis**

| shape | n | shape | n | shape | n | shape | n | shape | n | shape | n | shape | n | shape | n | shape | n | shape | n | shape | n | shape | n |
| --- | --- | --- | --- | --- | --- | --- | --- | --- | --- | --- | --- | --- | --- | --- | --- | --- | --- | --- | --- | --- | --- | --- | --- |
| 1 | 386 | 1 | 264 | 1 | 375 | 1 | 107 | 1 | 138 | 1 | 116 | 1 | 125 | 1 | 171 | 1 | 76 | 1 | 110 | 1 | 64 | 1 | 96 |
| 2 | 596 | 2 | 259 | 2 | 143 | 2 | 259 | 2 | 274 | 2 | 83 | 2 | 104 | 2 | 149 | 2 | 137 | 2 | 136 | 2 | 2 | 2 | 110 |
|  |  | 3 | 459 | 3 | 161 | 3 | 130 | 3 | 92 | 3 | 58 | 3 | 162 | 3 | 87 | 3 | 93 | 3 | 94 | 3 | 69 | 3 | 79 |
|  |  |  |  | 4 | 303 | 4 | 287 | 4 | 131 | 4 | 156 | 4 | 100 | 4 | 71 | 4 | 128 | 4 | 68 | 4 | 31 | 4 | 66 |
|  |  |  |  |  |  | 5 | 199 | 5 | 230 | 5 | 157 | 5 | 173 | 5 | 31 | 5 | 30 | 5 | 91 | 5 | 111 | 5 | 88 |
|  |  |  |  |  |  |  |  | 6 | 117 | 6 | 230 | 6 | 101 | 6 | 118 | 6 | 160 | 6 | 68 | 6 | 119 | 6 | 52 |
|  |  |  |  |  |  |  |  |  |  | 7 | 182 | 7 | 79 | 7 | 88 | 7 | 84 | 7 | 66 | 7 | 68 | 7 | 60 |
|  |  |  |  |  |  |  |  |  |  |  |  | 8 | 138 | 8 | 130 | 8 | 70 | 8 | 142 | 8 | 140 | 8 | 119 |
|  |  |  |  |  |  |  |  |  |  |  |  |  |  | 9 | 137 | 9 | 46 | 9 | 134 | 9 | 95 | 9 | 65 |
|  |  |  |  |  |  |  |  |  |  |  |  |  |  |  |  | 10 | 158 | 10 | 56 | 10 | 67 | 10 | 54 |
|  |  |  |  |  |  |  |  |  |  |  |  |  |  |  |  |  |  | 11 | 17 | 11 | 127 | 11 | 66 |
|  |  |  |  |  |  |  |  |  |  |  |  |  |  |  |  |  |  |  |  | 12 | 89 | 12 | 125 |
|  |  |  |  |  |  |  |  |  |  |  |  |  |  |  |  |  |  |  |  |  |  | 13 | 2 |
