# Supplementary material for: Association of GRM7 Variants with Different Phenotype Patterns of Age-Related Hearing Impairment in an Elderly Male Han Chinese Population
Source: PLoS One. 2013 Oct 11;8(10):e77153. doi: 10.1371/journal.pone.0077153 (PMC3795658; doi:10.1371/journal.pone.0077153)
Supplement: Table S4 — ANOVA table of mean square error and F statistic in the ARHI case subjects group. (DOC) [file pone.0077153.s004.doc]

**Table S4: ANOVA table of mean square error and F statistic in the ARHI case subjects group**

| frequency | 12shape | | 11shape | | 10shape | | 9shape | | 8shape | |
| --- | --- | --- | --- | --- | --- | --- | --- | --- | --- | --- |
|  | Error**^§^** | F^‡^ | Error^§^ | F^‡^ | Error^§^ | F^‡^ | Error^§^ | F^‡^ | Error^§^ | F^‡^ |
| 0.5khz | 30.96 | 39.90 | 33.54 | 32.93 | 33.46 | 36.84 | 32.75 | 44.84 | 33.95 | 44.39 |
| 1khz | 26.56 | 185.37 | 26.54 | 204.11 | 26.20 | 230.98 | 26.88 | 250.02 | 29.35 | 249.97 |
| 2khz | 41.40 | 306.57 | 40.66 | 345.01 | 43.94 | 346.54 | 44.39 | 384.57 | 48.04 | 395.42 |
| 4khz | 41.42 | 485.78 | 42.08 | 524.41 | 43.64 | 557.78 | 44.26 | 616.92 | 44.63 | 698.00 |
| 6khz | 28.91 | 730.98 | 26.90 | 871.60 | 27.17 | 957.55 | 29.89 | 967.94 | 32.80 | 995.56 |
| 8khz | 42.82 | 581.86 | 46.05 | 588.14 | 50.63 | 584.57 | 55.35 | 591.11 | 56.90 | 653.22 |

§Mean square, ‡F statistic.
